# Pycnodysostosis: a case series of eight Saudi patients with cathepsin K gene mutation and a literature review

**DOI:** 10.3389/fendo.2025.1517840

**Published:** 2025-04-17

**Authors:** Afaf Alsagheir, Raghad Alhuthil, Ahmad T. Alissa, Faisal Joueidi, Ahmed G. Sayed, Waleed Al-Amoudi, Alanoud S. Alabdulhadi, Bassam Bin-Abbas

**Affiliations:** ^1^ Department of Pediatrics, King Faisal Specialist Hospital and Research Centre, Riyadh, Saudi Arabia; ^2^ College of Medicine, Alfaisal University, Riyadh, Saudi Arabia

**Keywords:** *CTSK* gene, osteopetrosis, skeletal deformities, osteomyelitis, bone fracture

## Abstract

Pycnodysostosis, a rare osteopetrosis subtype, is mainly caused by homozygous or compound heterozygous biallelic pathogenic mutation of the cathepsin K (*CTSK*) gene. The cohort included eight patients (four males and four females) with a mean current age of 13 years (SD ± 3.6) and a mean age at diagnosis of 5 years (SD ± 2). All patients had a positive family history of pycnodysostosis and were born to consanguineous parents. Genetic analysis revealed that all individuals carried the same mutation: *NM_000396.3(CTSK):c.244-29A>G*. Clinically, they exhibited characteristic craniofacial features and skeletal deformities consistent with the diagnosis. Bone fractures were reported in 7 out of 8 patients, highlighting a significant clinical burden. All affected individuals received growth hormone therapy(GHT), though response to treatment varied among the group. These findings emphasize the importance of early genetic screening, particularly in families with a known history of pycnodysostosis, to enable timely diagnosis and intervention. Although pycnodysostosis is typically described as a nonprogressive skeletal dysplasia, the presence of complications such as osteomyelitis and recurrent fractures may contribute to a more complex and progressive clinical course in some patients.

## 1 Introduction

Pycnodysostosis, a rare osteopetrosis subtype, was first described by Albers-Schönberg in the 20th century (1). It is mainly caused by a homozygous or compound of heterozygous biallelic pathogenic mutation of the cathepsin K (*CTSK*) gene (2). *CTSK* is a membrane papain-cysteine protease family that encodes polypeptide chain 329 amino acids in chromosome 1q2 (3). It contains a three-dimensional structure with typical papain-like folds comprising L and R domains, with a cleft between the catalytic sites (4). *CTSK* forms a molecular complex with glycosaminoglycans, representing the collagenolytically active form of protease. In the presence of chondroitin sulfate, this complex arranges into bead-like structures along a strand-like organization that activates the substrate binding site (4). Highly expressive in osteoclasts, *CTSK* regulates bone resorption and osteoclast remodeling (3). Although the pycnodysostosis incidence rate is unknown, studies suggest approximately 1–1.7 cases per million births, with high consanguinity rates contributing to the disease’s etiological manifestation (5).

The clinical features of pycnodysostosis present with various systemic skeletal and dental abnormalities, including short stature, facial dysmorphology, maxillary and mandibular hypoplasia, polydactyly, and brachydactyly (1, 6). Truncal deformities comprise scoliosis, kyphosis, and recurrent chest infections (6). Dental features include delayed eruption of permanent teeth, retained deciduous dentition, malposed teeth, narrow and grooved palate, enamel hypoplasia, and abnormal tooth morphology (2, 5). Other systemic manifestations include laryngomalacia, stridor, sleep apnea, and dental manifestations (1). Although pycnodysostosis pathophysiology remains unclarified, studies suggest the involvement of a genetic enzyme encoding *CTSK, which* disrupts and impairs normal osteoclast-mediated bone resorption, resulting in osteosclerosis (2, 5). Low levels or deficiency of *CTSK* causes alterations in lysosomal cysteine protease activity, which can decrease osteoclast expression and impair bone turnover. This disruption affects bone resorption and remodeling, potentially leading to systemic osteosclerosis (3, 5, 7).

Pycnodysostosis diagnosis is established during infancy based on detailed history and physical examination, clinical presentation, radiological investigations, and genetic analysis (8). Its management is nonspecific. However, conservative management such as growth hormones, a multidisciplinary approach including psychiatrists, orthopedic surgeons, and pediatricians, and genetic early screening in families with a history of pycnodysostosis is essential to establish better outcomes (2, 8, 9). The disease is considered nonprogressive in nature, but several complications, including osteomyelitis and bone fracture, may alter the progressiveness of such cases (2).

## 2 Methods

This retrospective case series research involved eight cases of pycnodysostosis currently receiving care at endocrinology clinics at King Faisal Specialist Hospital and Research Center (KFSHRC) in Riyadh, Saudi Arabia. Data retrieval occurred in December 2023 from our electronic records medical system and included both pediatric and adult patients. Individuals without available genetic testing data were excluded. The study documented patients’ demographics, medical history, presentations, management, and investigative results.

Genetic testing was conducted as part of routine clinical practice using DNA extraction from peripheral blood samples, and whole-exome sequencing was carried out at the Molecular Diagnostic Laboratory of the Clinical Genomic Department Center for Genomic Medicine at KFSHRC.

This study was cleared and approved by the Ethics Committee at King Faisal Specialist Hospital and Research Center (No. 2245443). A waiver of consent was granted from the ethics committee, given the retrospective nature of the study.

## 3 Results

Patient’s demographics and presentations are summarized in **Tables 1**, **2**.

**Table 1:** Demographics & genetic test information.

#	Family	Sex	Current Age	Age at diagnosis	Region	Mutation	ACMG classification	Zygosity	Family history	Consanguinity
1	Fam-1	M	12 years	6 years	AlDamam	NM_000396.3(CTSK):c.244-29A>G	LP	Homo	+ve	yes
2	Fam-1	M	17 years	7 years	AlDamam	NM_000396.3(CTSK):c.244-29A>G	LP	Homo	+ve	yes
3	Fam-2	F	15 years	3 years	Alqassim, Buridah	NM_000396.3(CTSK):c.244-29A>G	LP	Homo	+ve	yes
4	Fam-3	M	12 years	3 years	Alqassim, Buridah	NM_000396.3(CTSK):c.244-29A>G	LP	Homo	+ve	yes
5	Fam-4	F	7 years	4 years	Almadinah	NM_000396.3(CTSK):c.244-29A>G	LP	Homo	+ve	yes
6	Fam-4	F	14 years	7 years	Almadinah	NM_000396.3(CTSK):c.244-29A>G	LP	Homo	+ve	yes
7	Fam-4	F	16 years	3 years	Almadinah	NM_000396.3(CTSK):c.244-29A>G	LP	Homo	+ve	yes
8	Fam-5	M	16 years	8 years	Almadinah	NM_000396.3(CTSK):c.244-29A>G	LP	Homo	+ve	yes

M, male; F, female; ACMG, American College of Medical Genetics, LP, Likely pathogenic; +ve, positive; Homo, Homozygous.

**Table 2:** Presentations.

#	Family	Deformities	Distinctive facial features	Fractures	Pseudotumor cerbri	Cognitive development	Clavicular dysplasia	Ostesclorosis	BMD z-score if done
1	Fam-1	yes	yes	yes	no	normal	no	yes	not done
2	Fam-1	yes	yes	yes	no	normal	yes	yes	not done
3	Fam-2	yes	yes	yes	yes, treated with acetazolamide	normal	not documented	yes	not done
4	Fam-3	yes	yes	yes	no	normal	no	yes	2.4
5	Fam-4	yes	yes	yes	not documented	normal	not documented	yes	not done
6	Fam-4	yes	yes	No	yes	normal	no	yes	not done
7	Fam-4	yes	yes	yes	no	normal	no	yes	not done
8	Fam-5	yes	yes	yes	no	normal	yes	yes	3.7

### 3.1 Case 1

A 12-year-old male, referred as a case of pycnodysostosis at the age of six, presented with short stature. At baseline, his height was 102.8 cm (SD −3.17) and his weight was 16.5 kg. His medical history was notable for a congenital heart defect—ventricular septal defect—which was discovered incidentally and surgically corrected. He was born at full term via spontaneous vaginal delivery and exhibited normal cognitive development and age-appropriate developmental milestones. On general examination, the patient was alert and oriented. Craniofacial and skeletal examination revealed dysmorphic features including frontal prominence, prominent eyes, blue sclera, and loosening of the mandibular angle. He also had short, broad hands and fingers, irregular and crowded teeth, and pectus carinatum. The remainder of his systemic examination was unremarkable. Laboratory investigations showed a serum calcium of 2.56 mmol/L, phosphate of 1.24 mmol/L, alkaline phosphatase of 237 IU/L, and a baseline IGF-1 of 120 ng/mL (as shown in **Table 3**). A full skeletal survey demonstrated features consistent with sclerotic dysplasia, including delayed skeletal maturation and diffuse osteosclerosis. Skull X-ray confirmed loosening of the mandibular angle, while spinal imaging showed a generalized increase in vertebral bone density. Radiographs of the pelvis and lower limbs showed diffuse metaphyseal sclerosis, and X-rays of the upper limbs revealed terminal phalangeal hypoplasia. A chest X-ray demonstrated bilateral clavicular hypoplasia. Following the confirmation of pycnodysostosis based on clinical, radiological, and genetic test, the patient was given a trial of recombinant human growth hormone (rhGH) therapy at a dose of 35 mcg/kg/day. Despite good adherence, there was no significant growth response to therapy (see **Table 4**). At final follow-up, his height reached 125.5 cm (SD −2.79) with a weight of 25.3 kg.

**Table 3:** Growth and biochemical data.

#	Family	Baseline height cm	Baseline height SDS	Last height cm	Last height SDS	growth velocity	ALP (Ref.: 100-300 U/L	Ca (Ref.: 2.10-2.60 mmol/L)	Phosphate (Ref.: 1.00-1.75 mmol/L)	1GF1 ng/mL
1	Fam-1	102.8	-3.17	125.5*	-3.79	4	237	1.24	0.87	120
2	Fam-1	92	−3.19	152*	-3.15	6	267	2.44	1.58	100
3	Fam-2	92.2	-1.59	141*	-3.3	5.6	280.1	2.34	1.52	135
4	Fam-3	85.3	-2.79	132.5	-2.65	6.72	278	2.45	1.75	123
5	Fam-4	95	-5.03	95	-5.6	not documented	not documented	not documented	not documented	not documented
6	Fam-4	102.5	-5.16	118.4*	-4.67	5.6	146.4	2.36	1.67	108
7	Fam-4	76	-5.74	110*	-8.2	2.5	1,280	2.23	0.65	106
8	Fam-5	107	-4.5	146.5*	-3.61	4.7	139.7	2.32	1.34	166

AL, alkaline phosphate; SDS, standard deviation score; Ref., Reference range.

*Final height.

**Table 4:** Management and outcome.

#	Family	Growth hormone therapy, dose	Compliant to treatment	Treatment response	Complications
1	Fam-1	Yes, 35 mcg/kg/day	yes	poor response	no
2	Fam-1	Yes, 35 mcg/kg/day	yes	poor response	no
3	Fam-2	Yes, 35 mcg/kg/day	yes	poor response	no
4	Fam-3	Yes, 35 mcg/kg/day	yes	poor response	Bone pain, knee pain, headaches, and attacks of sleep apnea
5	Fam-4	Yes, 35 mcg/kg/day	Discontinued After 3 months		Discontinued GH by family, due to side effect (headache).
6	Fam-4	Yes, 35 mcg/kg/day	yes	poor response	no
7	Fam-4	Yes, 35 mcg/kg/day	yes	poor response	no
8	Fam-5	Yes, 35 mcg/kg/day	yes	Beneficial	hip pain, stopped for two weeks then continued

### 3.2 Case 2

A 17-year-old male was referred for evaluation of short stature and growth failure. He was previously diagnosed with pycnodysostosis at the age of seven and had a history of obstructive lung disease, for which he was being treated with montelukast (Singulair) and fluticasone/salmeterol (Seretide). At presentation, his baseline height was 92 cm (SD −3.19) and his weight was 13.4 kg. He was born at full term via spontaneous vaginal delivery and demonstrated normal cognitive development with appropriate developmental milestones. On general examination, the patient was alert and oriented. Head and neck examination revealed dysmorphic features, while the remainder of the systemic examination was unremarkable. Laboratory investigations revealed a calcium level of 2.44 mmol/L, phosphate 1.58 mmol/L, alkaline phosphatase 267.1 IU/L, and a baseline IGF-1 of 100 ng/mL (refer to **Table 3**). Radiological evaluation revealed normal bone age without overt osteosclerosis. A skull X-ray demonstrated loosening of the mandibular angle and dolichocephaly. Chest imaging revealed generalized increased bone density and hypoplasia of the lateral aspects of the clavicles. A pelvic X-ray also showed generalized osteosclerosis. X-rays of the upper and lower limbs showed hypoplasia and fragmentation of the distal phalanges, particularly involving the right thumb, middle, and ring fingers, as well as the left thumb and middle finger. Additionally, an old, healed fracture was noted in the diaphysis of the right tibia, with evidence of partial periosteal and cortical callus formation. Following confirmation of the diagnosis, the patient was given a trial of recombinant human growth hormone (rhGH) therapy at a dose of 35 mcg/kg/day. He demonstrated good compliance and a positive response to treatment, with an observed growth velocity of 2.3 cm/year. At final follow-up, his height had reached 152 cm (SD −3.15).

### 3.3 Case 3

A 15-year-old female presented with short stature, skeletal dysplasia, and a 4-month history of headaches. Her medical history was significant for multiple recurrent midshaft fractures of the left tibia (three episodes) and a diagnosis of pseudotumor cerebri at age seven, for which she was treated with acetazolamide (Diamox) 125 mg. She was delivered at term via spontaneous vaginal delivery and had normal cognitive development and achievement of developmental milestones. On physical examination her baseline, height was 92.2 cm (SD −1.59) and weight 11.6 kg, she was alert and oriented. Ophthalmologic evaluation revealed papilledema, while the remainder of her systemic examination was unremarkable. Laboratory findings showed: Calcium: 2.34 mmol/L, Phosphate: 1.52 mmol/L, Alkaline phosphatase: 280.1 IU/L, baseline IGF-1: 135 ng/ml. Imaging studies revealed characteristic skeletal abnormalities consistent with a diagnosis of pycnodysostosis. Skull X-ray demonstrated generalized increased bone density, the presence of intrasutural wormian bones, hypotelorism, and an obtuse mandibular angle. Spinal imaging identified a pars interarticularis defect at the L5–S1 level. Hand and foot X-rays showed acro-osteolysis involving the distal phalanges, consistent with distal tuft resorption. Based on the constellation of clinical presentation, biochemical parameters, and radiological findings, a diagnosis of pycnodysostosis was confirmed. The patient was started on recombinant human growth hormone (rhGH) therapy at a dose of 35 mcg/kg/day. Despite documented adherence to treatment, no significant improvement in growth velocity was observed over the treatment period (see **Table 4**). Her pseudotumor cerebri resolved with acetazolamide therapy and notably did not recur following initiation of GHT. At her most recent follow-up, she had achieved a final height of 141 cm (SD −2.07).

### 3.4 Case 4

A 12-year-old boy was referred for evaluation of developmental delay and short stature. He was noted to have a large head, prompting a CT scan at a local hospital, which was reported as normal. At presentation, his height measured 85.3 cm (SD −2.79) and his weight was 13.5 kg. His past medical history included a diagnosis of pseudotumor cerebri at the age of ten, for which he was treated with acetazolamide 250 mg. The patient also reported ongoing bone and knee pain, frequent headaches, and episodes of sleep apnea. Despite these complaints, his cognitive development was appropriate for age, and he began walking at 15 months. However, a speech delay was noted during early childhood. On physical examination, the patient was alert and oriented. Head and neck examination revealed multiple dysmorphic features, including macrocephaly, a prominent nose, absence of the mandibular angle, grooved and fragile nails, wrinkled skin, brachydactyly, an open anterior fontanelle, and crowded teeth. The remainder of the systemic examination was unremarkable. Laboratory evaluation showed a calcium level of 2.45 mmol/L, phosphate level of 1.75 mmol/L, alkaline phosphatase of 278.1 IU/L, and a baseline IGF-1 of 123 ng/mL. Radiological imaging supported the clinical findings. Skull X-ray showed mild microcephaly with increased skull bone density and a persistently open anterior fontanelle. Spine imaging revealed a mild, diffuse increase in bone density involving the cervico-thoraco-lumbosacral vertebrae, along with bilateral hypoplastic clavicles. Pelvic and limb radiographs demonstrated a mild to moderate diffuse increase in bone density. Hand and foot X-rays revealed acro-osteolysis affecting the distal phalanges, and delayed ossification of the carpal bones was also noted. Based on the integration of clinical, biochemical, and radiographic data, a diagnosis of pycnodysostosis was established. The patient was started on recombinant human growth hormone therapy (rhGH) at a dose of 35 mcg/kg/day. Despite adherence to therapy, no significant improvement in linear growth was observed. Notably, the patient’s pseudotumor cerebri improved significantly following the initiation of with acetazolamide and has not recurred while on GHT.

### 3.5 Case 5

A 7-year-old female was referred to the outpatient clinic for evaluation of short stature and faltering growth. At presentation, her baseline height measured 95 cm (SD −5.03) and her weight was 13.75 kg. The patient had a history of multiple recurrent fractures, involving the scapula, tibia, and vertebrae. Family history was significant for first-degree parental consanguinity. She was born at term via spontaneous vaginal delivery, and her cognitive development and developmental milestones were within normal limits. On physical examination, the patient was alert and oriented. Craniofacial examination revealed several dysmorphic features, including frontal bossing, broad nasal bridge, hypertelorism, retrognathia, and brachycephaly. Other systemic examinations were unremarkable. Following confirmation of the diagnosis of pycnodysostosis, The patient was started on recombinant human growth hormone therapy (rhGH) at a dose of 35 mcg/kg/day. Although she was compliant with therapy, she developed headaches and was later diagnosed with atrial fibrillation during the course of treatment (see **Table 4**). These adverse events were considered potential side effects of therapy and prompted further clinical evaluation and monitoring.

### 3.6 Case 6

**Figure 1:**
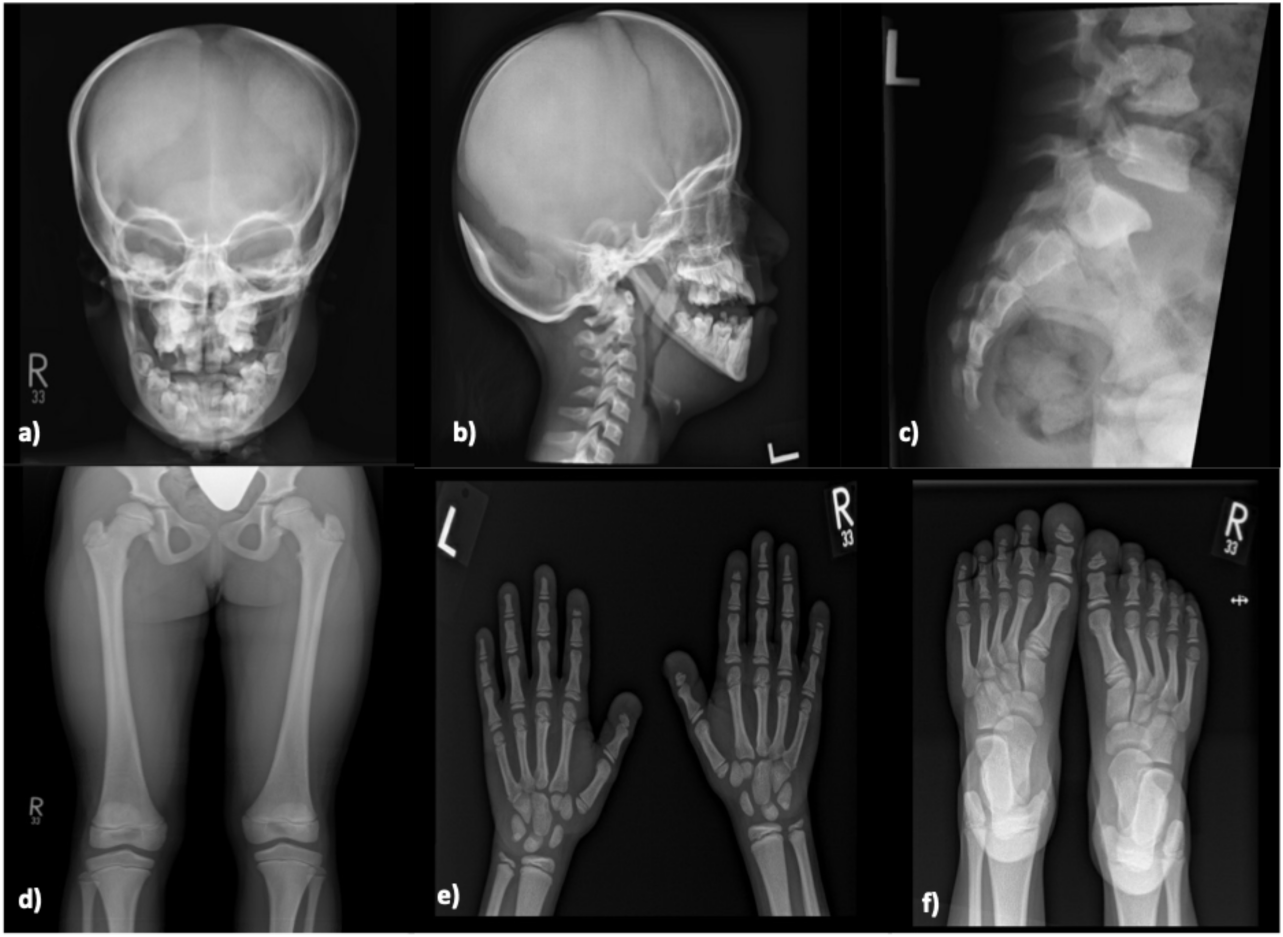
Skeletal X-ray of a male patient done at 11-year-old (Case 6) revealing moderate diffuse increased density of the skeletal bones with persistent open skull sutures, and widenings most prominent of the posterior sagittal and lambdoid sutures, anterior fontanelle, and straight mandible angles with abnormal teeth are noted **(a, b)**. Irregularity at the posterior elements of the C2, C3 are seen **(b)**. Spondylolysis /grade 1 spondylolisthesis of L5-S1 **(c)**. Mild to moderately hypoplastic bilateral acetabulae are noted, with 8 mm lateral uncoverage of the right femur head, 12 mm lateral uncoverage of the left femur head seen **(d)**. Prominent tufts resorptions /distal acro-osteolysis of bilateral feet and hands distal phalanges are noted **(e, f)**. Overall Sclerosis and dysplastic skeletal features are compatible with Pycnodysostosis.

A 14-year-old female presented to the pediatric endocrinology clinic with history of growth failure and short stature. At the time of presentation, her baseline height was 102.5 cm and weight was 16.1 kg. Her medical history was notable for chronic snoring and episodes of apnea during sleep, in addition to a prior diagnosis of pseudotumor cerebri at the age of 10 years, which had been managed with acetazolamide. Family history revealed first-degree consanguinity and a positive familial history of short stature. She was born full term via spontaneous vaginal delivery (SVD) and demonstrated normal cognitive development and timely achievement of developmental milestones. On physical examination, the patient was alert and oriented with craniofacial dysmorphic features. The remainder of her systemic examination was unremarkable. Laboratory investigations revealed calcium of 2.36 mmol/L, phosphate of 1.67 mmol/L, alkaline phosphatase of 146.4 IU/L, and a baseline IGF-1 level of 108 ng/mL (**Table 3**). Radiographic evaluation supported the clinical suspicion of skeletal dysplasia. Skull X-ray showed a widely persistent open suture line, most prominently involving the posterior sagittal and lambdoid sutures. Spinal imaging revealed grade 1 spondylolisthesis of L5/S1 with mild bilateral clavicular hypoplasia. Pelvic X-ray demonstrated generalized osteosclerosis, and X-rays of the upper and lower limbs showed hypoplasia of the distal phalanges, involving both thumbs and index fingers, as well as the left middle finger and right little finger (**Figure 1**).

Based on clinical, radiological, and genetic findings, a diagnosis of pycnodysostosis was established. The patient was started on recombinant human growth hormone therapy (rhGH) at a dose of 35 mcg/kg/day and no significant response to growth hormone therapy was noted. Her final recorded height was 118 cm (SD −4.67). Notably, her pseudotumor cerebri showed marked improvement with acetazolamide therapy, with no recurrence or worsening during the course of growth hormone treatment.

### 3.7 Case 7

A 16-year-old female presented to the pediatric clinic with severe short stature, growth retardation, and skeletal dysplasia. At the age of 3 years, her baseline height was 76 cm, and her weight was 8 kg. Her growth velocity was 2.5 cm per year. The patient had a history of recurrent fracture, neonatal diabetes mellitus (on-off hypoglycemic attacks), seizures, and was on insulin for three months, in addition to her menarche (irregular) having persistent hypophosphatemia on phosphate supplements. Neonatal history showed SVD at full term. The patient exhibited normal cognitive development with normal developmental milestones. On general examination, the patient was alert and oriented. Head and neck examination showed dysmorphic features including large ear, high arch palate, multiple oral ulcers, small face and hands, protruded mandible with midface deficiency, and moderate tenderness over the occipital area radiating the neck and upper back associated with blurred vision, which was suggestive of pseudotumor cerebri. Other systemic examinations were unremarkable. Laboratory investigations showed Ca: 2.23, phosphate: 0.65, alkaline phosphatase 1,280 and baseline IGF1 is 106 (**Table 3**). A skeletal survey revealed an increase in skeleton density, which was suggestive of osteopetrosis. A skull X-ray showed persistent opening in the sagittal suture with abnormal ossification of the calvarium and fusion of the coronal and lambdoid sutures with significant generalized osteopenia. A pelvic X-ray and x-rays of both limbs revealed bilateral subluxation of the hip joint with mild bilateral cox valga, generalized osteopenia, and prominent trabeculation in the bones. Erosion existed in the tuft of the terminal phalanges of the toes. After the diagnosis of pycnodysostosis, the patient’s treatment was initiated with human growth hormone therapy (rhGH) at a dose of 35 mcg/kg/day, but she responded poorly to the treatment and his pseudotumor cerebri showed clinical improvement and did not progress during the course of growth hormone therapy.

### 3.8 Case 8

**Figure 2:**
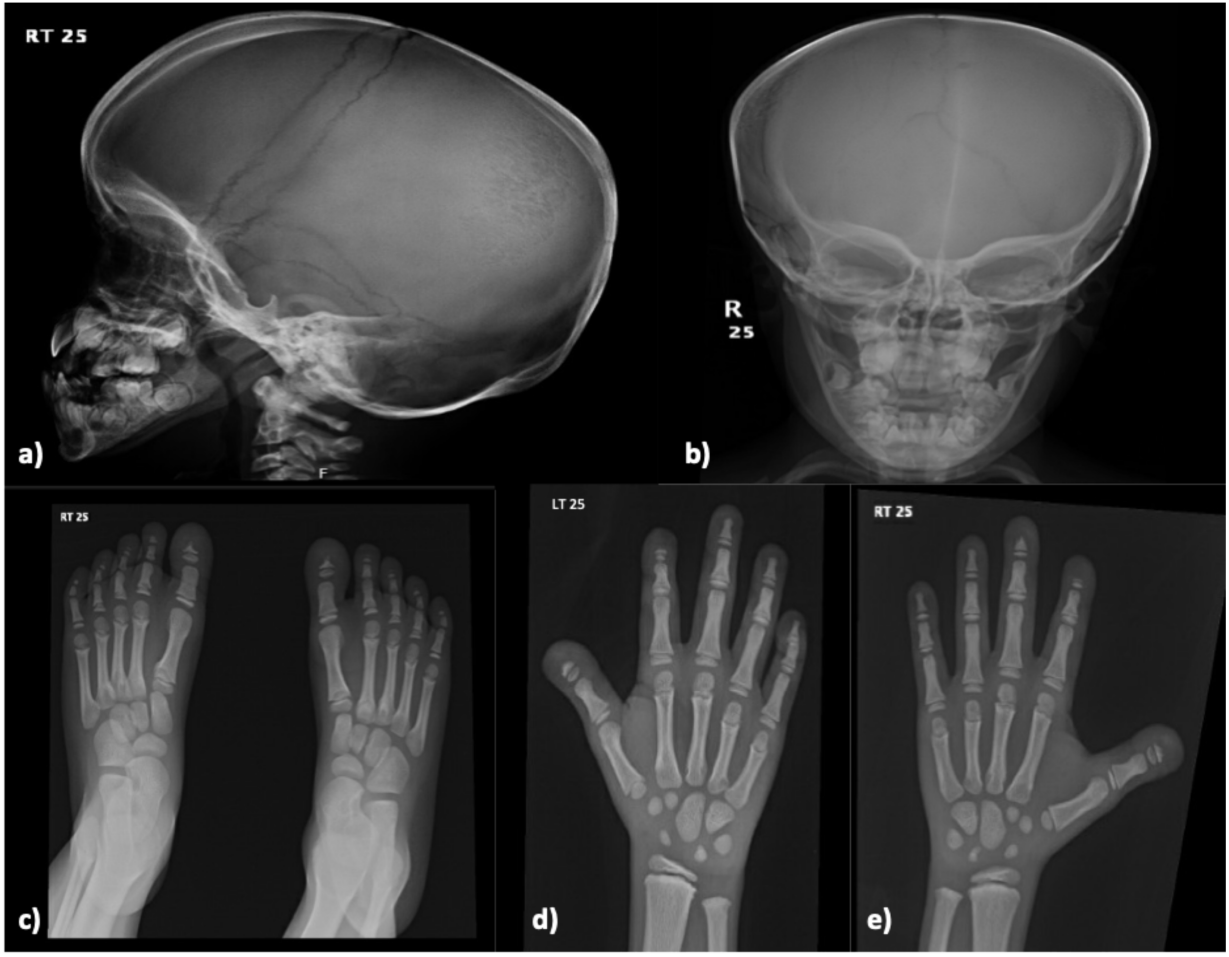
Skeletal X-ray of a male patient done at 8-year-old (Case 8) revealing multiple wormian bones identified in the skull with straightening of the mandibular angle **(a, b).** There are mild-to-moderate resorption of the distal phalanges of the hands and feet on both sides **(c–e)**. Findings are consistent with Pycnodysostosis.

**Figure 3:**
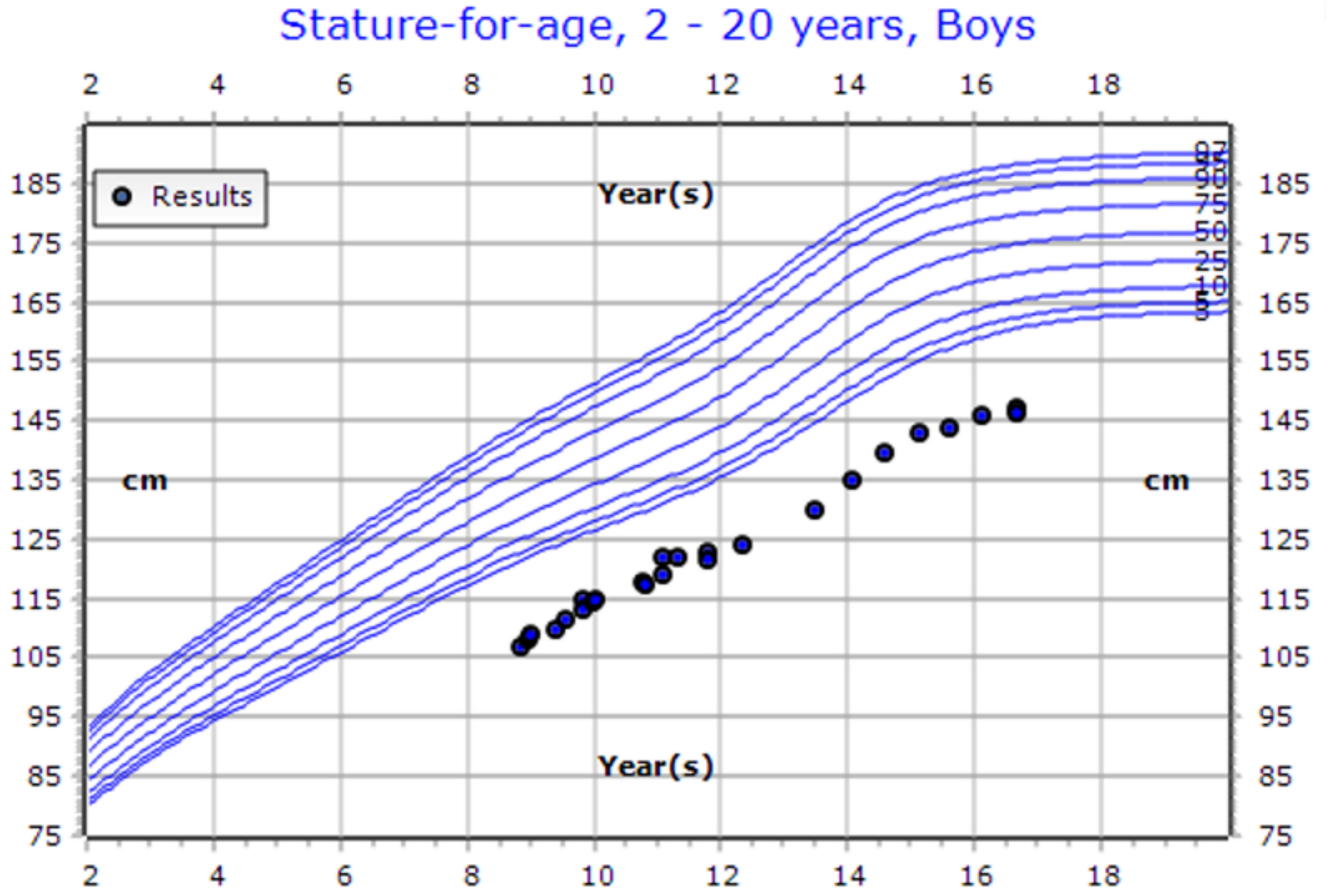
CDC Growth chart of a male patient (Case 8). At the presentation (aged 11 years old), his height measured 122 cm (SDS: -3.38, centile: 0.04). At his last visit (aged 16 years old), his height was recorded as 146.5 cm (SDS: -3.61, centile: 0.02). He is currently undergoing growth hormone therapy at a dose of 0.03 mg/kg/day.

A 16-year-old boy has been under follow-up in the pediatric endocrinology clinic for short stature since the age of two years. At baseline, his height was 107 cm (SD −4.5), weight was 16.5 kg, and growth velocity was 4.7 cm/year. His medical history included a left forearm fracture at the age of two years and a deviated nasal septum. Family history revealed first-degree parental consanguinity and two maternal uncles with a confirmed diagnosis of pycnodysostosis. He was born full term via spontaneous vaginal delivery and achieved normal developmental milestones with appropriate cognitive development. On general examination, the patient was alert and oriented. Head and neck examination was notable for moderate adenoid hypertrophy, with no overt dysmorphic features. Laboratory investigations demonstrated calcium of 2.32 mmol/L, phosphate of 1.34 mmol/L, alkaline phosphatase of 139.7 IU/L, and a baseline IGF-1 level of 166 ng/mL. A skeletal survey revealed a generalized increase in bone density, along with multiple wormian bones in the skull and straightening of the mandibular angle (**Figure 2**). Radiographs also showed mild to moderate resorption of the distal phalanges of both hands and feet, in association with features of skeletal dysplasia. Following a confirmed diagnosis of pycnodysostosis, the patient was initiated on recombinant human growth hormone (rhGH) therapy at a dose of 35 mcg/kg/day. He demonstrated good adherence to the treatment and responded favorably, with his height at the most recent visit reaching 146.5 cm (SD −3.61) and a height velocity of 3.1 cm/year (**Figure 3**). During treatment, he developed hip pain, which was managed conservatively.

## 4 Discussion

This single-center study presents the clinical, radiological, and genetic characteristics of eight patients diagnosed with pycnodysostosis and evaluates their response to growth hormone therapy (GHT). Given that the effectiveness of GHT remains a key predictor of clinical improvement, this analysis provides valuable insights into patient-specific outcomes and therapeutic responses.

The papain family of cysteine proteinases includes several phenotypes—B, H, L, S, and K—that are widely expressed in mammalian tissues. Among these, the K phenotype is encoded by the *CTSK* gene, which plays a critical role in bone resorption through its collagenolytic action on type I collagen molecules (10). The *CTSK* gene spans approximately 12 kb and comprises eight exons. To date, 63 mutations have been identified in *CTSK*, including frameshift, nonsense, missense, and splice-site mutations (6).

Among the reported pathogenic variants, *p.Gly180Ser* has been linked to disruption of cathepsin K activity due to its role in enzymatic structure and function. Similarly, mutations affecting the *p.Ala277* residue, located near the protein’s active site, are associated with severe phenotypic features. The splice-site mutation *c.244-29A>G*, identified in all patients in our cohort, appears to be regionally clustered, suggesting a potential founder effect (6). Additionally, the *p.Trp2Ter* mutation results in a premature termination codon, which significantly compromises protein function (6).

Functionally, cathepsin K is a major lysosomal cysteine protease in the bone matrix, essential for osteoclast-mediated bone resorption. Deficiency in this enzyme due to *CTSK* mutations leads to osteoclast dysfunction, contributing to the sclerotic and fragile bone phenotype observed in pycnodysostosis (10). The role of *CTSK* in bone homeostasis has also been supported by murine models, where its absence leads to impaired apoptosis of osteoclasts and an increased number of functionally compromised cells (14).

Pycnodysostosis was originally described by Maroteaux and Lamy in 1962 as a rare autosomal recessive skeletal dysplasia characterized by distinctive skeletal and dental anomalies (11, 12). The global prevalence is estimated at 1–1.7 cases per million, with a higher frequency reported in consanguineous populations (10, 13).

In our study, all eight patients were homozygous for the *c.244-29A>G* mutation. Clinically, all patients had a positive family history of pycnodysostosis, with most cases arising from first-degree consanguinity. Dysmorphic features, delayed skeletal maturation, and diffuse osteosclerosis were consistent findings. Seven patients experienced recurrent bone fractures, most commonly affecting the scapula, vertebrae, femur, and tibia. Clavicular hypoplasia or dysplasia was observed in five patients, acro-osteolysis in four, osteopetrosis in two, and dental anomalies in two.

Four patients developed pseudotumor cerebri, a known but poorly understood complication in pycnodysostosis, potentially resulting from persistently open cranial sutures and altered cerebrospinal fluid dynamics. While the exact pathophysiology remains unclear, regular ophthalmologic evaluation for signs of raised intracranial pressure is essential (20). For instance, Al Hashmi et al. reported a case of a 13-year-old with visual deterioration due to papilledema, managed successfully with acetazolamide and a ventriculoperitoneal shunt (20).

Radiologically, our findings were consistent with established features of pycnodysostosis, including generalized osteosclerosis, persistent open cranial sutures, clavicular hypoplasia, delayed ossification, and resorption of the distal phalanges (1, 15–17). Dental findings in the literature include enamel hypoplasia, mandibular hypoplasia, hypercementosis, and pulp chamber narrowing (9, 16, 18, 19).

Xue et al. found that short stature was present in 95.9% of patients and increased bone density in 88.7% (19). Similarly, fractures—particularly midshaft fractures of long bones—are among the most common complications, reported in nearly half of affected individuals (2, 15).

Genetic diagnosis is critical for confirming pycnodysostosis, enabling appropriate counseling and guiding management. Molecular testing not only confirms the clinical diagnosis but also allows for investigation of genotype-phenotype correlations. In addition to *CTSK*, genes such as *IL6R* and *MCL1*, located within the same chromosomal region, have been implicated in monocyte/macrophage differentiation and osteoclastogenesis, suggesting a broader genetic landscape influencing disease variability (13, 14).

The clinical features of pycnodysostosis span a range of skeletal and dental manifestations, including osteosclerosis, bone fragility, acro-osteolysis of the distal phalanges, frontal bossing, open fontanelles, mandibular retraction, clavicular anomalies, and skull deformities with delayed suture closure (1, 15–17). Dental abnormalities frequently include enamel hypoplasia, mandibular hypoplasia, and other defects (9, 16, 18, 19).

In our cohort, in addition to the core skeletal phenotype, four patients developed pseudotumor cerebri and one experienced persistent hypophosphatemia. The frequency and diversity of complications underscore the need for comprehensive evaluation and individualized care plans.

The diagnosis of pycnodysostosis relies on a combination of clinical, radiological, and molecular findings. Once confirmed, management must involve a multidisciplinary approach that includes pediatric endocrinology, orthopedics, dentistry, and genetics (21). Genetic counseling is particularly important for families from high-consanguinity backgrounds.

This study confirms that although the phenotypic presentation of pycnodysostosis is relatively consistent, the severity of complications and variability in response to treatment—particularly growth hormone therapy (GHT)—can be significant. GHT has demonstrated promising outcomes in managing growth challenges in pediatric patients with pycnodysostosis (22). Several studies have reported improvements in disproportionate growth, linear height gains, and a reduction in skeletal complications when GHT is administered (1).While the optimal timing for initiating GHT remains undefined, early treatment appears to mitigate disease progression and reduce the risk of fractures and other complications (23). Soliman et al. found that early GHT leads to increased IGF-1 secretion, enhances osteoclast differentiation, and promotes restoration of linear growth and skeletal proportions, helping patients achieve normal or near-normal stature (22). In contrast, delayed therapy often results in limited efficacy, underscoring the need for early evaluation and intervention in children presenting with reduced growth velocity (17, 22). A recent study reported that five out of six patients achieved a final height exceeding 150 cm following GHT. Similarly, Sulaiman et al. documented near-normal height (−2.25 SDS) after 18 months of therapy, with substantial improvement in long-bone growth and correction of skeletal disproportions, suggesting an excellent response to treatment (**Table 5**) (22). Despite these encouraging reports, our study demonstrated mixed outcomes. Among the eight patients treated with GHT at a dose of 35 mcg/kg/day, only three showed a positive response with improvements in height, bone density, and growth velocity. Four patients showed poor response and experienced adverse effects such as headache, bone pain, and sleep apnea. Three others showed no measurable response, leading to discontinuation or adjustment of therapy. These findings highlight the variability in GHT response, which may be influenced by the age of initiation, treatment duration, genotype differences, or hormonal sensitivity.

**Table 5:** A descriptive summary of reported pycnodysostosis cases in the literature.

Article	Age	Gender	Symptoms	Gene	Management	Complications
Khoja et al. (2015) (5)	13-year-old	Female	Short stature, euryprospic with deficient midface, frontal bossing, suture diastasis, absence of upper incisor with mispositioning of teeth,	–	Cemented upper bonded expander with midline screw expansion	Not reported
Verma et al. (2020) (8)	7-year-old	Female	Short stature, poor weight gain, dysmorphic nails, multiple dental caries and grooved palate, hepatoslenomegaly	Homozygous missense variant CTSK:C.890G>C.	Growth hormone 0.16 mg/kg/week, levothyroxine 50 µg/day	Not reported
Sulaiman & Thalange (2021) (22)	22-month-old	Male	Short stature, failure to thrive, jaundice gaping fontanelle, lumbar lordosis, short limbs	Homozygous frameshift mutation of CTSK gene in exon 4 (c.338delG)	Growth hormone 1 μg/kg/d; 0.5 mg	Not reported
Aynaou et al. (2016) (15)	12-year-old	Female	Short stature, multiple fractures of long bones, dysplastic nails, frontal and occipital bossing, groove palate, impacted and deciduose teeth, maxillary hypoplasia	–	Oral hygiene, psychiatric support	Not reported

Orthopedic management in pycnodysostosis poses additional challenges due to the inherent bone fragility and sclerotic architecture. Early surgical intervention, including internal plate fixation or intramedullary nailing, may be necessary to address fractures, deformities, or scoliosis and should be tailored to the patient’s condition (21). Hald et al. (1), in their review of 27 cases from France, reported a high frequency of fractures, surgical interventions, sleep apnea, and psychomotor delays, further emphasizing the need for GHT and multidisciplinary care, including mental health support (1).

Dental care is also essential. Although none of the patients in our cohort developed severe dental or maxillofacial complications, previous literature reports significant risks. Maintaining oral hygiene, implementing preventive dental strategies, and adopting careful procedural planning are critical due to the risk of mandibular and craniofacial complications (5, 18). For instance, Moroni et al. described a case of mandibular osteonecrosis in a patient with pycnodysostosis, highlighting the susceptibility of these individuals to severe dental and maxillofacial complications, particularly following dental interventions (18).

Thus, this study provides a comprehensive clinical, radiological, and genetic analysis of pycnodysostosis cases, but it has some limitations. The small sample size limits generalizability, and variation in treatment duration, adherence, and response to GHT restricts conclusions regarding its overall benefit. Future studies with larger cohorts and longer follow-up periods are needed to establish standardized protocols for treatment.

## 5 Conclusion

Pycnodysostosis is a rare skeletal dysplasia primarily associated with consanguineous populations and families. Recent advancements in our understanding have provided valuable insights into pathophysiology, management, and potential therapeutic targets. It requires prompt care from the specialized pediatric team. It is still an expandable entity that integrates complex mechanisms and requires prompt care, follow-up, genetic counseling, and careful observation for the patient’s family to reach optimal outcomes for the patient and future offspring. We recommend multidisciplinary approaches for clinical management alongside the contributions of colleagues, which is crucial for improving outcomes and quality of life.
